# Clinical efficacy of chemoembolization with simultaneous radiofrequency ablation for treatment of adrenal metastases from hepatocellular carcinoma

**DOI:** 10.1186/s40644-018-0157-5

**Published:** 2018-07-31

**Authors:** Hongjun Yuan, Fengyong Liu, Xin Li, Yang Guan, Maoqiang Wang

**Affiliations:** Department of Interventional Radiology, Chinese PLA General Hospital, 28 Fuxing Road, Beijing, 100853 People’s Republic of China

**Keywords:** Chemoembolization, Therapy, Radiofrequency ablation, Adrenal metastases

## Abstract

**Background:**

This study investigated the safety and efficacy of transcatheter arterial chemoembolization (TACE) with simultaneous radiofrequency ablation (RFA) as treatment for adrenal metastases (AM) from hepatocellular carcinoma(HCC).

**Methods:**

The records of 63 patients with AM who were treated at our Hospital between February 2013 and August 2016 were retrospectively reviewed. Patients were divided into a TACE+RFA group (*n* = 38) and a control group that received TACE alone (*n* = 25) according to different treatment methods. The success rate, tumor control rate, and safety of these groups were compared, and survival was evaluated using the Kaplan-Meier method.

**Results:**

All treatments could be completed technically successful in both groups. The tumor control rate at first imaging after 1 months was 92.1% (35/38) in the TACE+RFA group and 76.0% (19/25) in the TACE group(*P* = 0.041). The assisted local tumor control rate allowing repeated interventions in case of local recurrence was 70.0% (7/10) in the TACE+RFA group and 30.8% (4/13) in the TACE group (*P* = 0.039). During the follow up period, the TACE+RFA group had better survival than the TACE group at 1 year (92.1% vs. 88.0%), 2 years (73.7% vs. 64.0%), and 3 years (55.3% vs. 44.0%) (*P* = 0.040). The mean survival time was 26.8 ± 2.0 months (95% CI, 22.8–30.7) in the TACE+RFA group and 17.5 ± 2.2 months (95% CI, 13.1–21.8) in the TACE group.

**Conclusion:**

TACE+RFA led to better control of local disease progression and longer survival time than TACE alone in the treatment of AM from HCC. Although patients given TACE+RFA had more complications than those given TACE alone, these complications were easily managed.

## Background

Many primary malignancies metastasize to the adrenal gland, and the risk for adrenal metastasis is as high as 32–73% in some populations of tumor patients [1]. Adrenal metastases (AM) mainly originate from tumors of the lung, liver, kidney, gastrointestinal system, and pancreas, and metastases may be disseminated via hematogenous, lymphatic, or local infiltration [1, 2]. Most patients with AM have no evidence of adrenocortical or medulloadrenal dysfunction, and no clinical symptoms, such as pain. The diagnosis of AM is mainly based on imaging results because there are no specific blood markers for AM [3]. AM may be found in patients with hepatocellular carcinoma (HCC) who undergo regular MRI/CT imaging after treatment. To benefit patient survival, it is necessary to actively treat AMs while treating HCC. If AM are large or there are metastases to major organs, surgical intervention may not be feasible [4]. Chemotherapeutic agents may be given as palliative treatment. Radiotherapy is one of the most established therapies for AM, specially Stereotactic Body Radiation Therapy(SBRT)and cyber knife [5, 6]. However, patients who are unsuitable or unwilling to undergo surgery or radiotherapy may be given minimally invasive treatments such as chemoembolization or radiofrequency ablation (RFA) [6, 7]. In transcatheter adrenal arterial chemoembolization (TACE), high-dose chemotherapeutic agents and lipiodol are used to embolize vessels that supply the tumor, in an effort to kill or slow the growth tumor cells. RFA has been widely used in the treatment of liver tumors due to its minimally invasive nature and favorable safety profile, and is also recently used for treatment of adrenal tumors [6–9]. Most studies have focused on TACE or RFA alone, and few studies have examined the combined use of TACE with RFA.

In this study, we retrospectively compared patients who received TACE+RFA with patients who received RFA alone for the treatment of AM to evaluate the safety and efficacy of these treatment methods.

## Methods

### Study design

The Institutional Review Board approved this retrospective study in August 2017. Written informed consent was obtained from all patients. A total of 63 patients were diagnosed with AM from HCCs at our hospital between February 2013 and August 2016, and the location of the primary tumor was confirmed for each patient. The included patients received simultaneous TACE+RFA (*n* = 38) or TACE alone (*n* = 25). All patients received Computed tomography (CT) or magnetic resonance imaging (MRI) and pathological examinations before treatment. TACE and RFA were performed by the same clinician who had more than 20 years of experience in interventional therapy. One month after the initial treatment, patients received a follow-up CT or MRI exams and had follow-up evaluations every 3 months thereafter. Local tumor progression and survival were evaluated. If residual tumor was observed during a follow-up evaluation, re-treatment was performed.

### Patient characteristics

The inclusion criteria were: diagnosis of a primary tumor and confirmation of AM by imaging examinations; contraindication for surgical intervention due to liver or kidney dysfunction or other contradictions such as cardiac or lung dysfunction, and severe diabetes mellitus; refusal of surgical intervention; and no receipt of local chemotherapy or radiotherapy. The exclusion criteria were: coagulation dysfunction and platelet count below 30 × 10^9^/L; local infection or uncontrollable systemic infection; contraindication for interventional therapy due to severe liver, kidney, or cardiac dysfunction; and Eastern Cooperative Oncology Group (ECOG) score of 0 to 2. The pros and cons of interventional therapy were explained to all patients before treatment, and written informed consent was obtained before treatment. Table 1 shows the baseline characteristics of the 63 patients who received treatment.

**Table 1:** Baseline characteristics of patients with AM who received TACE+RFA or TACE alone

Variable	TACE+RFA	TACE	χ^2^/*t*	*P*
Age, years	54.2 ± 9.3	56.8 ± 8.8	1.108	0.272
Gender			0.092	0.762
M	26 (68.4%)	18 (72.0%)		
F	12 (31.6%)	7 (28.0%)		
Child-Pugh grade			0.076	0.783
A	30 (78.9%)	19 (76.0%)		
B	8 (21.1%)	6 (24.0%)		
ECOG			0.406	0.523
0	33 (86.8%)	23 (92.0%)		
1	5 (13.2%)	2 (8.0%)		
Outcome of primary tumor			0.834	0.933
Resection	9 (23.7%)	5 (20.0%)		
CR^a^	17 (44.7%)	12 (48.0%)		
PR	7 (18.4%)	5 (20.0%)		
SD	1 (2.6%)	0		
PD	4 (10.5%)	3 (12.0%)		
Extra-adrenal metastases			1.638	0.440
None	16 (42.1%)	7 (28.0%)		
One organ metastases	14 (36.8%)	13 (52.0%)		
Multiple organ metastases	8 (21.1%)	5 (20.0%)		
AM location			0.118	0.731
Unilateral	29 (76.3%)	20 (80.0%)		
Left	11 (28.9%)	11 (44.0%)		
Right	18 (47.4%)	9 (36.0%)		
Bilateral	9 (23.7%)	5 (20.0%)		
Maximal diameter of AM(cm)			0.414	0.519
≥ 3	11 (23.4%)	9 (30.0%)		
<3	36 (76.6%)	21 (70.0%)		
Tumor size of AM(cm)				
Range	1.5~ 7.3	1.2~ 8.1		
Mean	3.3 ± 1.6	3.5 ± 1.7	0.473	0.637

*Abbreviations*: *ECOG* Eastern Cooperative Oncology Group, *CR* complete remission, *PR* partial remission, *SD* stable disease, *PD* progressive disease, *AM* adrenal metastasis

Note ^a^Assessed according to the mRECIST

### Instruments

TACE and RFA were performed under the guidance of an Angio-CT (Miyabi; Siemens Medical Solutions AG, Erlangen, Germany), which combines Artis zee digital subtraction plate angiography and a Somatom Emotion 16-slice spiral CT (Siemens Medical Solutions AG, Erlangen, Germany). RFA was performed using a RITA radiofrequency system (model 1500;RITA Medical System, Mountain View, CA, USA) and a RITA electrode.

### Treatments

**Fig. 1:**
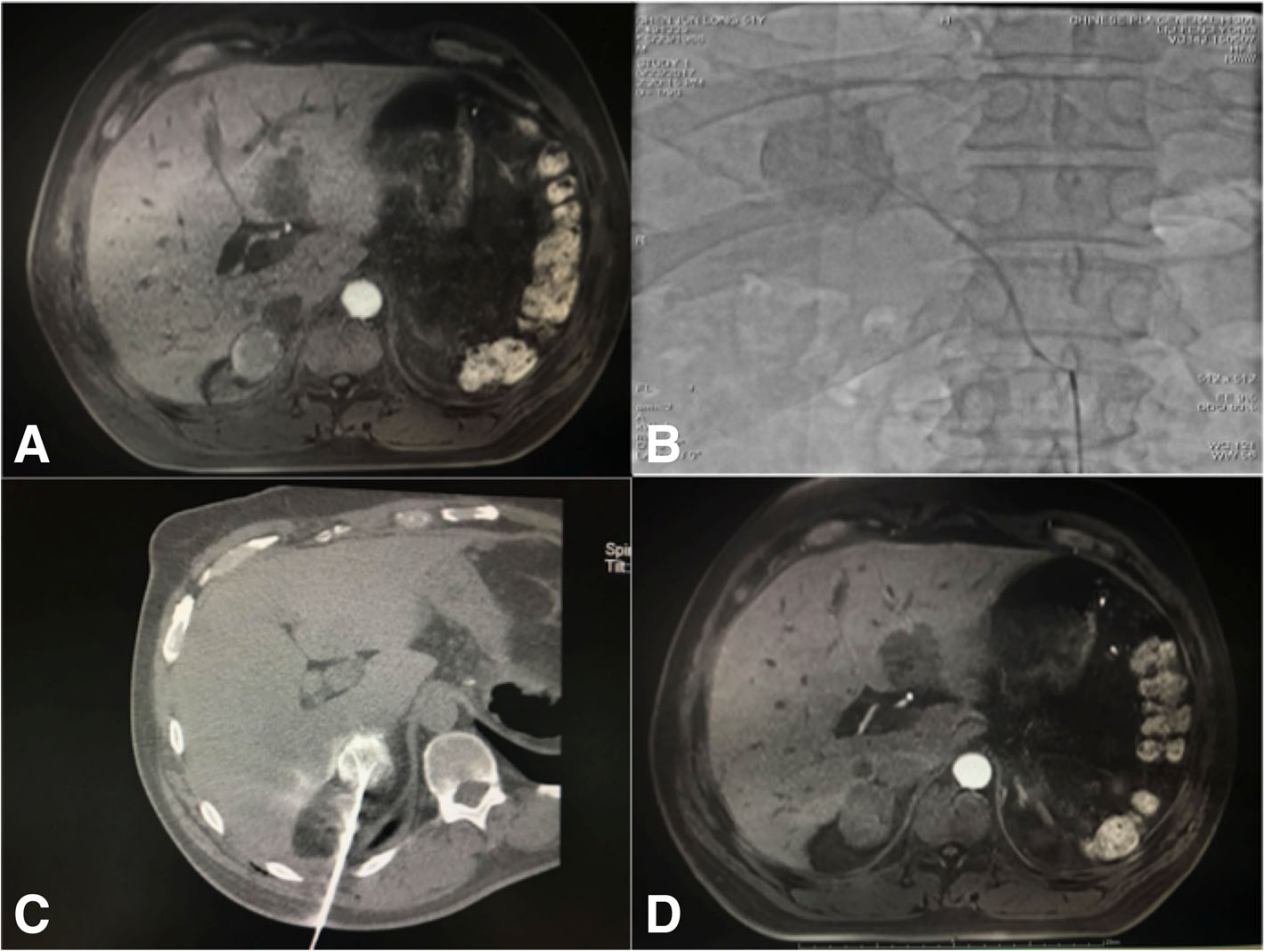
A 52-year-old male patient with right adrenal metastases from HCC who received TACE+RFA. **a** Enhanced MRI before surgery, indicating adrenal metastases with uneven enhancement. **b** TACE of the adrenal metastases. **c** RFA immediately after TACE. **d** Enhanced MRI 1 month after surgery, indicating no enhancement or coagulation necrosis

**Fig. 2:**
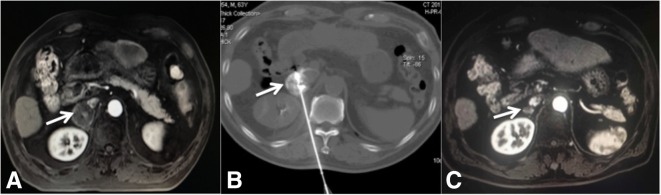
A 63-year-old male patient with right adrenal metastases from HCC who received TACE+RFA. **a** Pre-operative enhanced MRI, showing right adrenal metastases with uneven enhancement (arrow). **b** RFA immediately after TACE of the adrenal metastases. **c** Enhanced MRI 2 years after treatment, showing no enhancement and a smaller tumor (arrow)

**Fig. 3:**
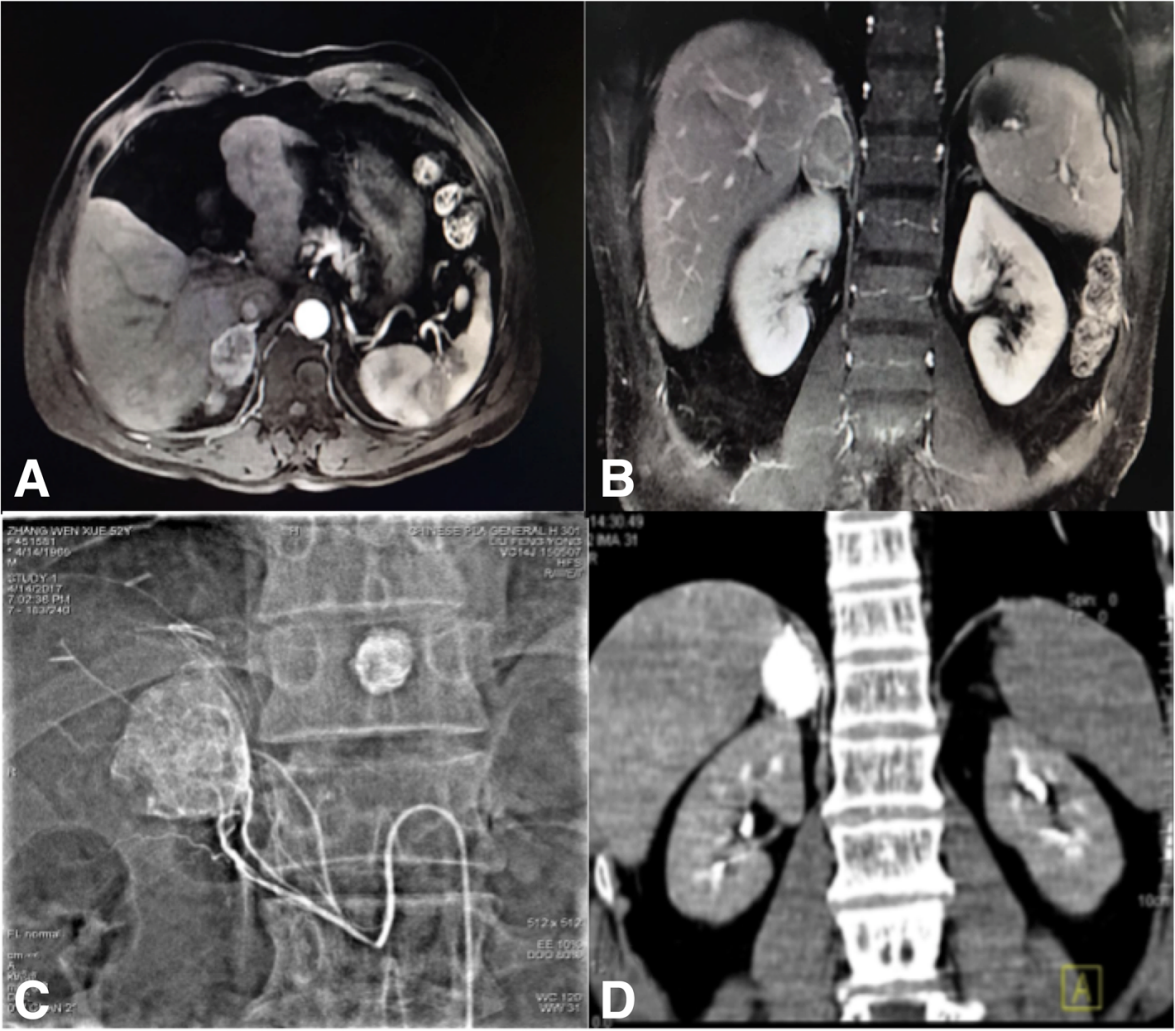
A 65-year old female patient with right adrenal metastasis of a primary liver tumor who received TACE alone. **a** & **b** Abdominal enhanced MRI, showing uneven enhancement in the arterial phase, and continuous enhancement in the venous phase. **c** TACE of the adrenal metastasis. **d** Abdominal CT at 1 month after treatment, showing favorable lipiodol deposition

Angio-CT combines digital subtraction angiography (DSA) and CT. For patients in the TACE+RFA group, TACE was conducted under the guidance of DSA, and then RFA was performed immediately afterwards under the guidance of CT (Figs. 1 and 2). Patients in the TACE group received TACE alone under the guidance of DSA (Fig. 3).

#### Pre-operative preparation

All patients received comprehensive preoperative examinations that included routine blood tests, detection of coagulation time, and measurement of kidney and liver function, plasma cortisol, and catecholamines. Intravenous access was established, and blood pressure was measured non-invasively. Blood oxygen saturation, pulse, and heart rate were monitored during the procedure, and oxygen was administered via a nasal catheter. Other emergency drugs and a ventilator were also prepared before treatment. Sodium nitroprusside, urapidil, and metoprolol were available for immediate administration during RFA.

#### TACE treatment

The TACE regimen was identical in the two groups. Each patient’s chemotherapeutic protocol was designed according to the primary tumor or pathology, and was prepared for administration by TACE after local sterilization and local anesthesia with 1% lidocaine. A modified Seldinger vascular puncture was performed at the femoral artery, and abdominal aortography was performed under the guidance of DSA (injection rate: 20 mL/s; total volume of contrast: 50–60 mL). The orifice of the adrenal artery was identified, and a 4 Fr Cobra catheter or Rosch hepatic (RH) catheter was used for selective angiography of the arteriae suprarenalis superior, arteriae suprarenalis media, and arteriae suprarenalis inferior to identify blood vessels supplying the tumor and their sources, sizes, and number. Then, a 2.6 Fr microcatheter (Progreat;Terumo Corp, Japan) was used for superselective catheterization of the supplying vessels, followed by chemoembolization. Epirubicin, mitomycin, and platinum-based chemotherapeutics were mixed with lipiodol to form an emulsion before periinterventional; 5-fluurouracil (5-FU), calcium levofolinate, and other chemotherapeutics were mixed with lipiodol, and the mixture was intermittently injected into the target vessels. During periinterventional, the embolization was strengthened with gelatin sponge particles or polyvinyl alcohol (PVA) particles when the target vessels were larger than the catheter; if the target vessels were small and tortuous, superselective catheterization could cause vascular spasm, so local injection of lidocaine before catheterization was performed. If the target vessels became spasmodic during the bolus injection of chemotherapeutics, this made the treatment impossible, so injection of the lipiodol emulsion was discontinued after relief of the vascular spasm, and lipiodol alone was injected to avoid chemotherapeutic-induced vascular spasm.

#### TACE+RFA treatment

For the TACE+RFA group, Patients were changed to the prone position after the TACE, and the C-arm was moved toward the dorsal side to leave a space for CT scanning. According to the preoperative CT or MRI results, the patient’s position was determined, and grid locators were placed. The CT parameters were 120 kV and 70 mA, and the slice thickness was 5 mm. After scanning, the puncture site, route, angle, and depth were determined according to the tumor size, location, adjacent structures, intra-operative lipiodol deposition, and peripheral status of the lesion. Then, surface markers were placed for the RFA. After skin sterilization, local anesthesia was performed using 1% lidocaine. The puncture needle was placed to avoid the liver, lungs, and major vessels, and CT was performed at least once to confirm its location. Once the needle reached the target, CT was performed again to confirm successful puncture, and this was followed by RFA. Tumor temperature and impedance were monitored in real time, and the output power was adjusted accordingly. The power was 140 to 200 W, the baseline impedance of the tumor was 50 to 70 Ω, and the treatment duration was 10 to 20 min. A multipole RF needle was used for ablation of lesions larger than 3 cm at multiple sites, until the periphery of ablation was 1 cm larger than the lesion size. A unipolar RF needle was used for ablation, except for lesions that were 3 cm or less from important organs. When the needle was withdrawn, ablation at 70–90 °C was performed at the puncture tract to prevent hemorrhage and possible implantation metastasis.

#### Intra-operative monitoring

Intra-operative monitoring was performed for both groups. In particular, vital signs (especially heart rate and blood pressure) were monitored closely because damage to the adrenal gland could cause release of large amounts of catecholamines, leading to sudden and significant increases in heart rate and blood pressure that could result in a life-threatening hypertensive crisis. If necessary, sodium nitroprusside or Betaloc was injected to reduce the blood pressure and heart rate.

#### Post-operative management

After treatment, both groups received analgesic therapy, anti-infection treatment, and urine alkalinization. The TACE+RFA group also received hydration therapy to improve the excretion of contrast and prevent renal injury. Vital signs were also closely monitored after interventions, and all side-effects and complications (such as pain, hemorrhage, fever, vomiting, hemoglobinuria, and hypertension) were recorded.

### Definitions and follow up

Technical success was defined as superselective catheterization of the supplying artery and puncture of the target lesion. One month after interventions, patients were followed up by CT or MRI, and the local lesion was assessed for evaluation of therapeutic efficacy. The extent of image-guided tumor ablation was defined according to the Standardization of Terminology and Reporting Criteria (2009 and 2014) [10, 11]. In particular, therapeutic efficacy was defined as lesion eradication, lesion shrinkage, or unchanged lesion without enhancement on CT or MRI; residual tumor was defined by enhancement of the lesion on CT or MRI (difference in CT of at least 20 HU; evident attenuation in MRI). Local tumor progression was defined as the appearance of tumor foci at the edge of the ablation zone, after at least one contrast-enhanced follow-up study documented adequate ablation, and the absence of viable tissue in the target tumor and surrounding ablation margin based on imaging. Survival time was defined as the time from first interventional adrenal treatment (TACE or TACE+RFA) to death or censoring (October 2017). The mean duration of follow up was 26.3 ± 19.3 months (range: 4–66 months). If residual tumor or tumor progression was observed during the follow up, retreatment could be performed.

### Statistical analysis

Statistical analysis was performed using SPSS version 24.0 (IBM Corporation, Armonk, NY, USA). Data are expressed as the mean ± standard deviations. A two-sample *t*-test was used to compare quantitative data between the two groups, and a chi square test was used for comparisons of qualitative data. Survival was analyzed using the Kaplan-Meier method. Curves for overall survival (OS) were compared using the log-rank test. A *p*-value below 0.05 was considered statistically significant.

## Results

### Technical success rate

All patients received superselective catheterization for chemoembolization. In the TACE+RFA group, one patient underwent a transhepatic approach and three patients received injections of normal saline to separate the adrenal tumor from adjacent structures, followed by puncture. All patients in both groups were treated successfully, and the technical success rate was 100%.

### Tumor control rate and local tumor progression

**Fig. 4:**
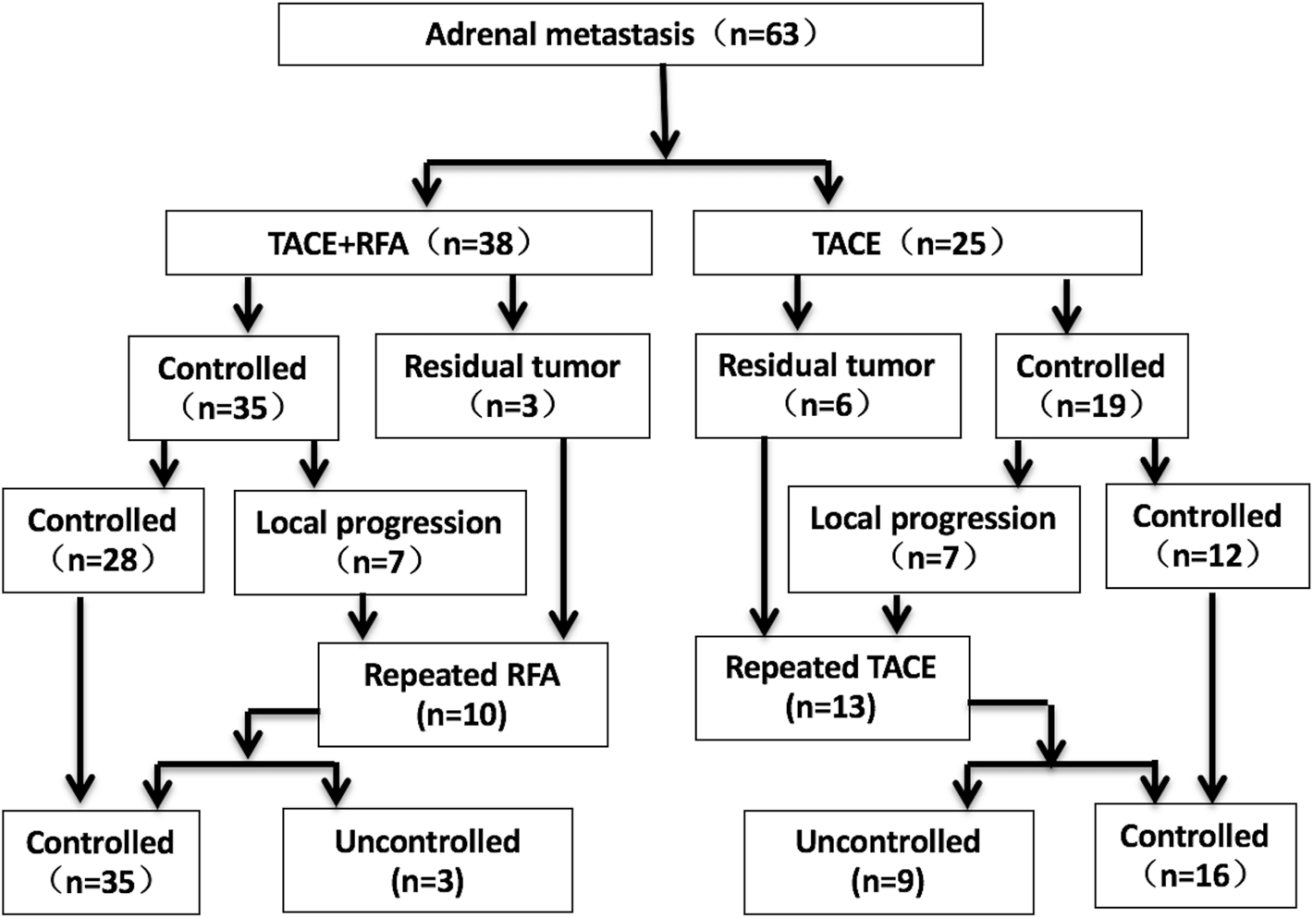
Outcomes of patients in the TACE+RFA and the TACE groups

**Fig. 5:**
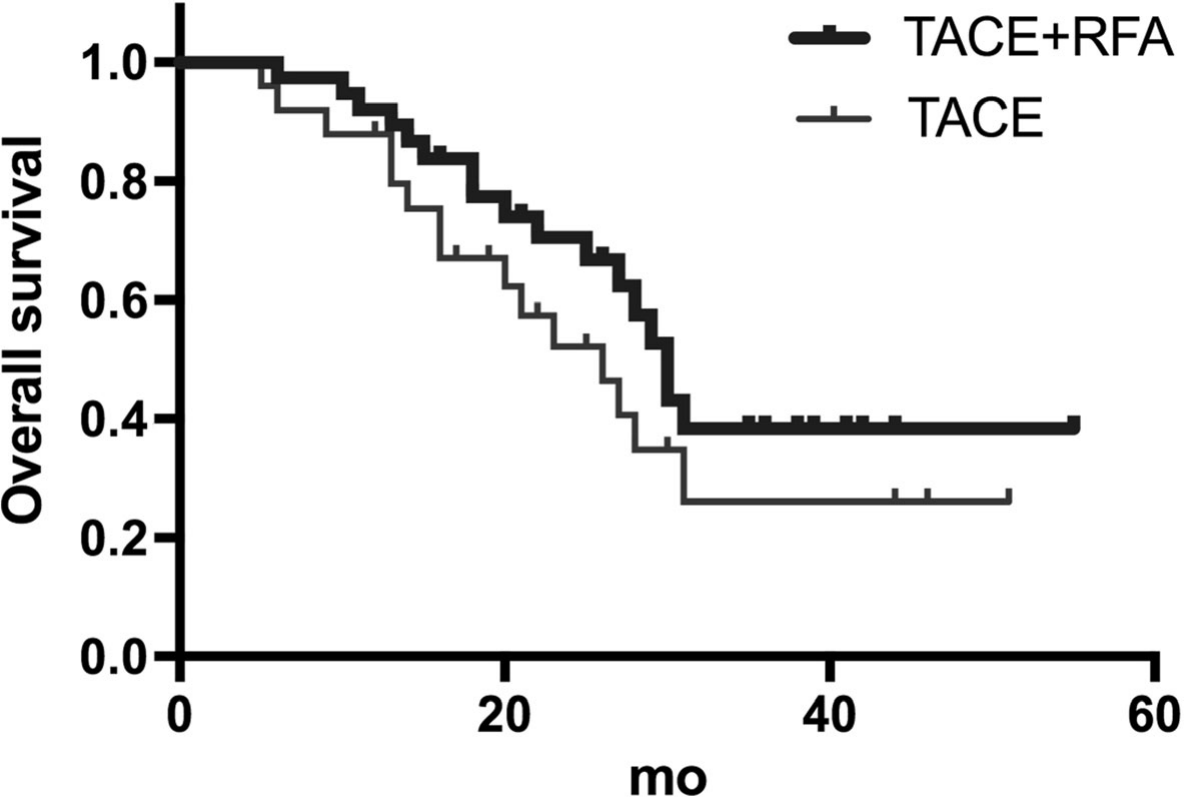
Overall survival in the TACE+RFA group (top) and the TACE group (bottom). A log-rank test(*P* = 0.041)indicated the difference of overall survival between the two groups is significant

The tumor control rate at first imaging after 1 months was 92.1% (35/38) in the TACE+RFA group and 76.0% (19/25) in the TACE group(*P* = 0.041). Three patients in the TACE+RFA group and 6 patients in the TACE group had residual tumors at the edge of the original tumors. At the end of follow up (Figs.4 and 5), 7 patients in the TACE+RFA group and 7 patients in the TACE group had local tumor progression. The assisted local tumor control rate allowing repeated interventions in case of local recurrence was 70.0% (7/10) in the TACE+RFA group and 30.8% (4/13) in the TACE group (*P* = 0.039). The mean local tumor progression-free survival was 8.6 ± 6.5mo in the TACE+RFA group and 6.4 ± 7.3 mo.

### Survival analysis

The overall survival rate was better for the TACE+RFA group than the TACE group at 1 year (92.1% vs. 88.0%), 2 years (73.7% vs. 64.0%), and 3 years (55.3% vs. 44.0%; *P* = 0.040). The mean survival time was 26.8 ± 2.0 months in the TACE+RFA group (95% CI, 22.8–30.7 months) and 17.5 ± 2.2 months in the TACE group (95% CI, 13.1–21.8 months).

### Complications and side effects

No patients in the TACE+RFA group had serious adverse effects after TACE (Table 2). However, 6 patients in the TACE+RFA group developed hypertensive crisis (blood pressure greater than 220/115 mmHg and heart rate greater than 117 beats/min) after RFA. In each case, ablation was immediately discontinued, a selective alpha-1 blocker was administered, blood pressure and heart rate normalized after 20 min, and ablation was then continued. Two patients in the TACE+RFA group developed mild hemothorax and pneumothorax. Pain was the most common adverse effect in both groups. Morphine was administered upon complaint of pain during surgery, and oxycodone hydrochloride was used as an analgesic after surgery. Twenty-one patients in the TACE+RFA group and 13 patients in the TACE group experienced post-embolization/post-ablation syndrome, characterized by fever (> 38.5 °C), nausea, vomiting, fatigue, and general malaise, and were given treatment to manage their symptoms. None of the patients died during surgery. The incidence of postoperative complications was higher in the TACE+RFA group than in the TACE group (presumably due to the RFA), all complications resolved after active management of symptoms, and no patients had life-threatening complications.

**Table 2:** Complications in the TACE+RFA and TACE groups

Complication	TACE+RFA	TACE
Hypertensive crisis	6	0
Hemothorax and pneumothorax	2	0
Post-embolization/post-ablation syndrome	21	13
Pain		
Mild(VAS ≤ 3)	16	10
Moderate(3<VAS ≤ 7)	10	5
Serious(7<VAS ≤ 10)	7	2

*Abbreviations VAS* visual analogue scale

## Discussion

Sorafenib is recommended as a first-line treatment to systemic treatment of hepatocellular carcinoma metastasis [12], but the effect of the adrenal metastasis of the HCC is rarely reported. In recent years, clinicians have increasingly used TACE and RFA for the treatment of AM due to the minimal invasiveness of these techniques. However, multiple treatment sessions are necessary to obtain complete tumor necrosis with RFA, and can greatly increase the risk of hypertensive crisis [13]. It is difficult to embolize all adrenal arteries because adrenal tumors are fed by several arteries [14, 15]. So we tried a combination treatment of RFA + TACE。The results of our study show that radiofrequency ablation combined with adrenal arterial chemoembolization is safe and effective management of adrenal metastasis of HCC.

Previous studies [16, 17] of patients with AM reported the disease control rate was 77–83% after local surgical intervention and the median survival time was 8–30 months, depending on the pathological type of the primary tumor. Specifically, the survival times was 11–29 months for patients with non-small cell lung tumor (NSCLC), 20–89 months for patients with renal cell carcinoma, 23–29 months for patients with colorectal tumor, and 12–21 months for patients with hepatocellular carcinoma (HCC) [16]. In our study, there was successful control of adrenal metastasis in 75.0% of patients in the TACE+RFA group and in 35.3% of patients in the TACE group at the last follow up evaluation. In addition, the mean survival time was 26.8 ± 2.0 months in the TACE+RFA group and 17.5 ± 2.2 months in the TACE group. The disease control rate and mean survival time after TACE+RFA were similar to those previously reported after adrenalectomy. This finding may be ascribed to a synergistic interaction of TACE and RFA. Wang et al. [18, 19] speculated that complete lipiodol deposition could provide better heat transduction when RFA is given immediately after TACE, and that this heat had a strong tumoricidal effect, especially for irregular tumors, because RFA alone cannot completely cover the whole lesion in all three dimensions. In other words, angio-CT guided TACE and RFA can transfer heat to surrounding tissues, thereby reducing recurrence and metastasis. Moreover, adrenal artery embolism may also have an anti-tumor effect because it blocks blood flow, reducing the cooling effect of circulation during RFA and decreasing heat loss [20]. In addition, TACE+RFA targets the lesion when the concentration of chemotherapeutics is the highest, possibly providing a synergistic anti-tumor effect [21]. We speculate that TACE+RFA increases the efficacy of treatment due to a synergistic interaction of TACE and RFA.

Our results also indicate that patients given TACE+RFA had better prognosis than those given TACE alone at 6 months (100% vs. 88.2%), 12 months (87.5% vs. 70.6%), and 24 months (68.8% vs. 41.2%). The overall survival rate was significantly better in the TACE+RFA group than in the TACE group. These results suggest the combined therapy is more effective. Yamakado et al. [22] investigated 6 patients with adrenal metastasis from HCC who received adrenal artery chemoembolization and RFA, and reported the median survival time was 24.9 months, similar to that of patients given adrenalectomy.

Six patients in our TACE+RFA group developed transient hypertension during RFA, and required a selective alpha-1 blocker (urapidil) and sodium nitroprusside to lower blood pressure. Urapidil and sodium nitroprusside are commonly used to control peri-operative hypertensive crises. Chini et al. [23] also reported that transient use of a heart-selective beta blocker (esmolol) effectively controlled RFA-induced hypertensive crisis. In addition, the TACE+RFA group had a higher incidence of post-embolization or post-ablation syndrome, a higher incidence of postoperative pain, and more severe post-operative pain. Considering the severity of postoperative pain after TACE+RFA, we recommend analgesic therapy for 3 days. Our results also showed that the incidences of complications and adverse effects were higher in the TACE+RFA group, presumably due to the use of RFA. However, these complications and adverse effects resolved after active management of symptoms, and no patients had severe complications. These findings indicate that TACE+RFA therapy is safe.

There were some limitations in this study. There might have been a selection bias caused by non-randomization. This was a single-center study, the sample size was relatively small, and some of the patients did not have biopsies and pathologic examinations, thus there is a possibility of misdiagnosis from the exclusive use of CT and MRI.

## Conclusions

Taken together, our results indicate that TACE+RFA is a feasible and effective treatment for AM from HCC. Although the use of concomitant RFA may increase post-operative complications and adverse effects rate, the complications and adverse effects were shown to resolve after active management of symptoms. Combined TACE+RFA treatment may prolong the survival time and benefit patients with AM from HCC.

## Abbreviations

AM: Adrenal metastasis
CR: Complete remission
ECOG: Eastern Cooperative Oncology Group
HCC: Hepatocellular carcinoma
PD: Progressive disease
PR: Partial remission
RFA: Radiofrequency ablation
SD: Stable disease
TACE: Transcatheter arterial chemoembolization
VAS: Visual analogue scale

## References

[1] Wagnerova H, Lazurova I, Felsoci M (2013). Adrenal metastases [J]. Bratislava Medical Journal-bratislavske Lekarske Listy.

[2] Lombardi CP, Raffaelli M, De CC (2006). Role of laparoscopy in the management of adrenal malignancies [J]. J Surg Oncol.

[3] Rajaratnam A, Waugh J (2005). Adrenal metastases of malignant melanoma: characteristic computed tomography appearances [J]. Australas Radiol.

[4] Sancho JJ, Triponez F, Montet X (2012). Surgical management of adrenal metastases [J]. Langenbecks Archives of Surgery.

[5] Ahmed KA, Barney BM, Macdonald OK (2013). Stereotactic body radiotherapy in the treatment of adrenal metastases [J]. Am J Clin Oncol.

[6] Desai A, Rai H, Haas JA, et al. A retrospective review of CyberKnife stereotactic body radiotherapy for adrenal tumors (primary and metastatic): Winthrop University hospital experience [J]. Front Oncol. 2015:185–5. 10.3389/fonc.2015.00185 PMC4538288 26347852

[7] Frenk NE, Daye D, Tuncali K (2017). Local control and survival after image-guided percutaneous ablation of adrenal metastases [J]. J Vasc Interv Radiol.

[8] Szejnfeld D, Nunes TF, Giordano EE (2015). Radiofrequency ablation of functioning adrenal adenomas: preliminary clinical and laboratory findings [J]. J Vasc Interv Radiol.

[9] Yamakado K, Anai H, Takaki H, et al. Adrenal metastasis from hepatocellular carcinoma: radiofrequency ablation combined with adrenal arterial chemoembolization in six patients [J]. Am J Roentgenol. 2009;192(6) 10.2214/AJR.08.1752 19457793

[10] Ahmed M, Solbiati L, Brace CL (2014). Image-guided tumor ablation: standardization of terminology and reporting criteria—a 10-year update [J]. Radiology.

[11] Ahmed M (2014). Image-guided tumor ablation: standardization of terminology and reporting criteria-a 10-year update: supplement to the consensus document [J]. J. Vasc. Interv. Radiol.

[12] Jordi B, Morris S (2011). Management of hepatocellular carcinoma: an update [J]. Hepatology (Baltimore, Md).

[13] Haga H, Saito T, Okumoto K (2005). Successful percutaneous radiofrequency ablation of adrenal metastasis from hepatocellular carcinoma [J]. J Gastroenterol.

[14] Park JS, Yoon DS, Kim KS (2007). What is the best treatment modality for adrenal metastasis from hepatocellular carcinoma? [J]. J Surg Oncol.

[15] Momoi H, Shimahara Y, Terajima H (2002). Management of adrenal metastasis from hepatocellular carcinoma [J]. Surg Today.

[16] Kim SH, Brennan MF, Russo P (2015). The role of surgery in the treatment of clinically isolated adrenal metastasis [J]. Cancer.

[17] Bradley CT, Strong VE (2014). Surgical management of adrenal metastases [J]. J Surg Oncol.

[18] Wang ZJ, Wang MQ, Duan F (2013). Clinical application of transcatheter arterial chemoembolization combined with synchronous C-arm cone-beam CT guided radiofrequency ablation in treatment of large hepatocellular carcinoma [J]. Asian Pac J Cancer Prev.

[19] Wang ZJ, Wang MQ, Duan F (2013). Transcatheter arterial chemoembolization followed by immediate radiofrequency ablation for large solitary hepatocellular carcinomas [J]. World J Gastroenterol.

[20] Yasumoto T, Hayashi S, Shimizu J (2009). [Radiofrequency ablation combined with transcatheter arterial chemoembolization for the local recurrent tumor after resection of the adrenal metastasis from hepatocellular carcinoma--a case report] [J]. Gan to Kagaku Ryoho Cancer & Chemotherapy.

[21] Wood BJ, Abraham J, Hvizda JL (2003). Radiofrequency ablation of adrenal tumors and adrenocortical carcinoma metastases [J]. Cancer.

[22] Hasegawa T, Yamakado K, Nakatsuka A (2015). Unresectable adrenal metastases: clinical outcomes of radiofrequency ablation [J]. Radiology.

[23] Chini EN, Brown MJ, Farrell MA (2004). Hypertensive crisis in a patient undergoing percutaneous radiofrequency ablation of an adrenal mass under general anesthesia [J]. Anesth Analg.

